# Association of Myopia With Microvascular Alterations in Patients With Type 2 Diabetes: An Optical Coherence Tomography Angiography Study

**DOI:** 10.3389/fmed.2021.715074

**Published:** 2021-10-01

**Authors:** Xin Wen, Zijing Li, Jianhui Xiao, Xuane Liu, Yichi Zhang, Yuqing Lan

**Affiliations:** Guangdong Provincial Key Laboratory of Malignant Tumor Epigenetics and Gene Regulation, Department of Ophthalmology, Sun Yat-sen Memorial Hospital, Sun Yat-sen University, Guangzhou, China

**Keywords:** microvascular alterations, myopia, diabetic retinopathy, axial length, optical coherence tomography angiography, vessel density

## Abstract

**Purpose:** To determine the association of myopia with peripapillary and macular microvasculature in eyes with type 2 diabetes using optical coherence tomography angiography (OCTA).

**Methods:** Diabetic patients with and without diabetic retinopathy (DR) were recruited and grouped according to myopic status in this cross-sectional study. Axial length, refractive error, and OCTA parameters were measured. OCTA parameters were analyzed with adjustment of confounding factors and further Bonferroni analysis was performed to determine the differences in multiple group comparisons.

**Results:** Compared with the diabetic eyes without myopia, those with myopia had lower rate of DR (21.82 vs. 35.90%, χ^2^ = 6.190, *P* = 0.013), longer axial lengths (24.94 ± 0.75 vs. 23.16 ± 0.64, *F* = 311.055, *P* < 0.001) and reduced whole vessel density (VD) of optic nerve head (ONH) (45.89 ± 5.76 vs. 49.14 ± 4.33, *F* = 19.052, *P* < 0.001), peripapillary VD (48.75 ± 6.56 vs. 50.76 ± 4.51, *F* = 7.600, *P* = 0.006), and reduced thickness of the retinal nerve fiber layer (RNFL) (95.50 ± 12.35 vs. 100.67 ± 13.68, *F* = 5.020, *P* = 0.026). In eyes without myopia, the superficial vessel density (SVD) (46.58 ± 4.90 vs. 43.01 ± 4.25; 95% CI, 1.80–4.61; *P* < 0.001), deep vessel density (DVD) (45.64 ± 6.34 vs. 42.15 ± 6.31; 95% CI, 1.07–5.00; *P* < 0.001), and FD300 area density (50.31 ± 5.74 vs. 44.95 ± 6.96; 95% CI, 2.88–7.27; *P* < 0.001) were significant reduced in eyes with DR(DR eyes) comparing to those without DR (NoDR eyes). In eyes with myopia, only SVD were significantly reduced in DR eyes comparing to NoDR eyes (41.68 ± 3.34 vs. 45.99 ± 4.17; 95% CI, 1.10–7.22; *P* = 0.002). In NoDR eyes, both whole VD of ONH and Peripapillary VD demonstrated a significant decrease in eyes with myopia comparing to those without myopia (49.91 ± 4.36 vs. 45.61 ± 6.32; 95% CI, 1.95–6.27; *P* < 0.001 and 51.36 ± 4.24 vs. 48.52 ± 6.99; 95% CI, 0.56–5.11; *P* = 0.006, respectively).

**Conclusions:** In diabetic patients, myopic eyes exhibited lower prevalence of DR and thinner thickness of RNFL. The refractive status could possibly impact the retinal microvascular changes from NoDR to DR stage.

## Introduction

Diabetic retinopathy (DR) was a leading global cause of preventable blindness in working-age population (1). Various clinical studies showed that myopia was a protective factor against the onset and progression of DR (2–4). The preventive effect was closely related to the degree of DR, axial length, and refractive error. The speculated cause was that the elongation of axial length thinned the retina or retinal nerve fiber layer (RNFL) and enlarge the peripapillary atrophy (5), resulting in reduced retinal oxygen demand and lower risk of DR. Nevertheless, the specific mechanism remained unknown. Using optical coherence tomography angiography (OCTA), a recently introduced non-invasive vascular imaging technology, it was possible to detect microvascular alterations layer by layer in retinal pathologies such as diabetes and myopia (6, 7). Despite conflicting results of myopia on the retinal vessel density (VD), myopia appeared to decrease the VD in either the macula or the optic nerve head (ONH), or in both regions in healthy adults (8–10). Because decreased VD in macula and ONH had also been reported in DR (11–14), the actual blood perfusion in diabetic patients with myopia was ambiguous. To our knowledge, quantitative OCTA analysis of microvasculature in diabetic patients with and without myopia had not yet been reported. Therefore, in the present study, we analyzed the OCTA parameters in diabetic eyes with and without myopia in order to determine the correlation between myopia and microvasculature in diabetic patients.

## Materials and Methods

### Participants

This cross-sectional retrospective study recruited type 2 diabetes mellitus (T2DM) patients with and without myopia from September 1, 2018 to September 1, 2019. The inclusion criteria were diagnosis of T2DM and disease duration >1 year. Exclusion criteria included ocular diseases with severe opacity of refractive media that would influence fundus examination or any other ocular diseases that would affect retinal circulation, including glaucoma, age-related macular degeneration, and retinal artery/vein occlusion. Because DR could cause cataract and affect the results of refractive error, and DR of any stage were closely related to mild myopia (15), for the purpose of minimizing the interference, we defined myopia as a spherical equivalent of < -3.0 diopters(D) or axial length of >24.00 mm and non-myopic eyes as a spherical equivalent of −2.75 to 0.5 D, with axial length of <24.00 mm (16).

### Clinical Examinations

All participants underwent comprehensive ophthalmic examinations, including best-corrected visual acuity (BCVA) (LogMAR visual acuity), intraocular pressure (IOP), slit-lamp biomicroscopy, and dilated fundus examination using a non-contact slit lamp lens, color fundus photographs, OCTA, and fundus fluorescein angiography (only in patients with DR).

### OCTA

The OCTA images were obtained using an RTVue XR Avanti spectral domain OCT device (Optovue, Inc., Fremont, CA, USA). A 3 × 3 mm area of macula and a 4.5 × 4.5 mm area of ONH were scanned at 70,000 A-scans/second with a wavelength of 840 nm. A split-spectrum amplitude-decorrelation angiography algorithm was used to detect erythrocyte movement in OCTA. The retinal vascular networks and nerve fibers of macula and ONH were analyzed.OCTA images with scan quality ≥ 6 were included. OCTA images with poor quality or large motion artifacts or shadow artifacts or segmentation errors were excluded.

### OCTA Image Analysis

Using prototype AngioVue software 2.0, the parameters of the macula and ONH were automatically measured and analyzed without any attempts at manual alteration. Macula-associated parameters included whole VD of superficial capillary plexus (SCP) and deep capillary plexus (DCP), foveal vessel density in a 300-μm region around FAZ (FD300). VD were defined as the percentage of the area occupied by blood vessels. The area of vessels was calculated using software by extracting a binary image from the grayscale OCTA image, and then calculating the percentage of pixels occupied by blood vessels in the defined region. The SCP slab was segmented from ILM to inner plexiform layer (IPL). The DCP slab was segmented from 15 μm posterior to the IPL to the outer plexiform layer (OPL). Other macula-associated parameters such as FAZ perimeter (PERIM), acircularity index (AI), foveal VD, parafoveal VD, and perifoveal VD in the SCP and DCP were not included, because the high correlation between any two of the above parameters would interfere the results of the statistical model. ONH-associated parameters included VD of the whole 4.5 × 4.5 mm area, peripapillary area as well as the average RNFL thickness of the entire peripapillary area. The peripapillary area was defined as a 1.0 mm-wide round annulus extending from the 2.0-mm circle of ONH.

### Statistical Analysis

SPSS 22.0 (SPSS Inc. Chicago, IL, USA) were used for statistical analyses. All variables were calculated as means ± standard deviation. Categorical variables were analyzed using the Chi-square test. OCTA parameters were analyzed using univariate general linear analysis and further Bonferroni analysis was used to assess the differences in multiple group comparisons. Analysis of OCTA parameters were performed with adjustment for confounding factors including age and signal strength (SS) of OCTA images. *P* < 0.05 were considered statistically significant.

## Results

### Study Population

We enrolled 250 eyes of 156 patients, including 55 eyes (15 bilateral eyes, 25 unilateral eyes including 10 right eyes and 15 left eyes) with myopia of 40 diabetic patients (mean ± SD age, 53.66 ± 12.58 years) and 195 eyes (79 bilateral eyes, 37 unilateral eyes, including 22 right eyes and 15 left eyes) without myopia of 116 diabetic patients (mean ± SD age, 49.09 ± 17.21 years). The myopic group included 43 eyes without DR and 12 eyes with DR. The non-myopic group included 125 eyes without DR and 70 eyes with DR. There were significant differences between the myopic and non-myopic groups with respect to age, gender, axial length, spherical equivalent refractive error, and the rate of DR (Table 1).

**Table 1:** Clinical and demographic characteristics of non-myopic and myopic eyes.

**Characteristics**	**No. (%) or mean** **±** **SD**			
	**Non-myopic (*n* = 195)**	**Myopic eyes (*n* = 55)**	***F*/**χ**^2^**	***P*-value**
Age, y (range)	53.66 ± 12.58 (18 to 89)	49.09 ± 17.21 (25–85)	4.761	0.030^*^
Female	86(86/195, 44.10%)	15(15/55, 27.27%)	4.372	0.037^*^
Axial length, mm (range)	23.16 ± 0.64 (21.61–24.00)	24.94 ± 0.75 (24.11–27.40)	311.055	<0.001^*^
Signal strength of OCTA images	7.33 ± 1.11 (6–10)	7.07 ± 1.02 (6–9)	2.356	0.126
Refractive error, D (range)	−0.88 ± 0.70 (−2.75 to 0.50)	−2.76 ± 2.70 (−12.00 to −0.25)	20.227	<0.001^*^
BCVA logMAR (range)	0.09 ± 0.41 (−0.20 to 4.00)	0.09 ± 0.30 (−0.10 to 1.20)	<0.001	0.992
IOP, mmHg (range)	15.57 ± 2.78 (11–26)	16.09 ± 2.61 (11–23)	1.520	0.219
Duration of DM, y (range)	6.10 ± 3.13 (1–15)	6.33 ± 2.66 (2–15)	0.236	0.628
HbA1c, % (range)	8.65 ± 1.59 (5.90–14.30)	8.19 ± 1.75 (6.20–15.40)	3.397	0.067
DR	70 (70/195, 35.90%)	12 (12/55, 21.82%)	6.190	0.013^*^
Mild NPDR	6 (6/70, 8.57%)	1 (1/12, 8.33%)	1.360	0.715
Moderate NPDR	50 (50/70, 71.43%)	9 (9/12, 75.00%)		
Severe NPDR	12 (12/70, 17.14%)	1 (1/12, 8.33%)		
PDR	2 (2/70, 2.86%)	1 (1/12, 8.33%)		

*BCVA, best corrected visual acuity; IOP, intraocular pressure; HbA1c, glycosylated hemoglobin. DR, diabetic retinopathy; NPDR, non-proliferative diabetic retinopathy*.

* *Indicates statistically significant difference between the groups. P < 0.05 were considered statistically significant*.

**Figure 1:**
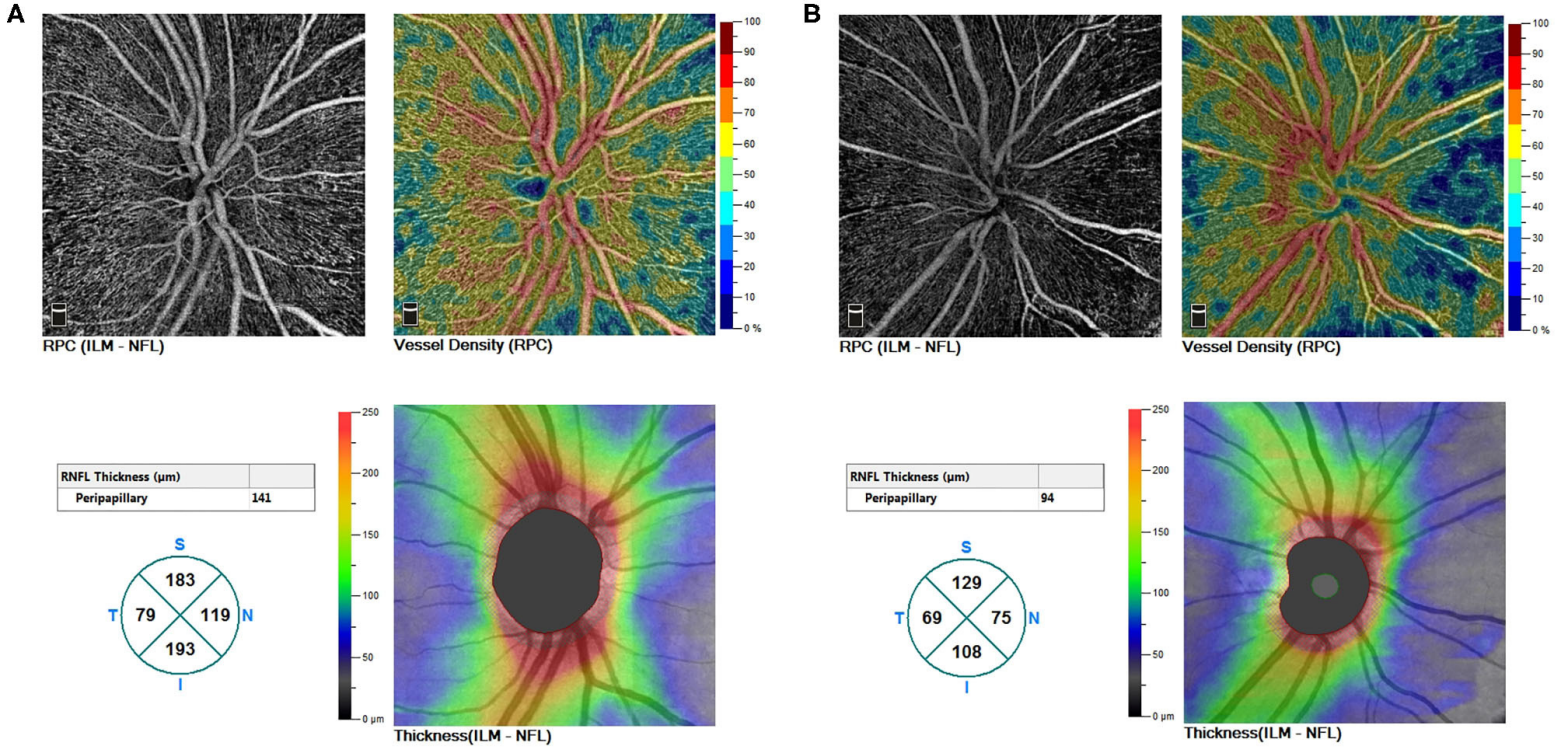
**(A)** A non-myopic eye without DR (a right eye of a male patient aged 47). **(B)** A myopic eye without DR (a right eye of a male patient aged 21). Whole VD of ONH, peripapillary VD, and thickness of RNFL demonstrated a decreased trend from non-myopic to myopic eyes. DR, diabetic retinopathy; VD, vessel density; ONH, optic nerve head; RNFL, the retinal nerve fiber layer.

The comparison of the RNFL and the VD between eyes of diabetic patients with and without myopia were displayed in Table 2. Reduced thickness of the RNFL were observed in myopic eyes comparing to non-myopic ones(95.50 ± 12.35 vs. 100.67 ± 13.68, *F* = 5.020, *P* = 0.026), especially in the nasal (68.56 ± 10.27 vs. 75.04 ± 16.11, *F* = 6.037, *P* = 0.015) and inferior (119.97 ± 23.28 vs. 127.65 ± 20.52, *F* = 4.298, *P* = 0.039) sections. Accordingly, diabetic eyes with myopia exhibited decreased whole VD of ONH (45.89 ± 5.76 vs. 49.14 ± 4.33, *F* = 19.052, *P* < 0.001) and peripapillary VD (48.75 ± 6.56 vs. 50.76 ± 4.51, *F* = 7.600, *P* = 0.006), comparing to eyes without myopia (Figure 1). However, no significant differences were found in terms of macula-associated parameters such as SVD, DVD, or FD300 area density between diabetic eyes with and without myopia (Table 2).

**Table 2:** Differences of RNFL and VD between non-myopic and myopic eyes of diabetes.

	**Non-myopic eyes (*n* = 195)**	**Myopic eyes (*n* = 55)**	***F***	***P*-value**
Thickness of RNFL (μm) (range)	100.67 ± 13.68 (40.80–148.00)	95.50 ± 12.35 (63.70–116.90)	5.020	0.026^*^
Superior (μm)	121.72 ± 20.85 (34.30–180.00)	120.43 ± 19.71 (66.50–155.10)	0.132	0.716
Nasal (μm)	75.04 ± 16.11 (43.60–163.50)	68.56 ± 10.27 (45.10–85.10)	6.037	0.015^*^
Inferior (μm)	127.65 ± 20.52 (34.00–217.10)	119.97 ± 23.28 (51.80–206.50)	4.298	0.039^*^
Temporal (μm)	77.23 ± 13.47 (36.80–131.20)	73.81 ± 12.85 (39.80–95.80)	3.054	0.082
Whole VD of ONH (%) (range)	49.14 ± 4.33 (36.97–65.65)	45.89 ± 5.76 (29.71–53.05)	19.052	<0.001^*^
Peripapillary VD (%) (range)	50.76 ± 4.51 (33.43–61.39)	48.75 ± 6.56 (28.15–58.44)	7.600	0.006^*^
SVD (%) (range)	45.30 ± 4.97 (29.34–55.87)	45.05 ± 4.36 (35.19–53.24)	0.037	0.847
DVD (%) (range)	44.38 ± 6.53 (18.21–62.83)	44.61 ± 6.16 (31.05–59.96)	0.956	0.329
FD300 area density (%) (range)	48.39 ± 6.70 (27.06–60.74)	48.88 ± 7.19 (30.80–61.93)	1.448	0.230

*RNFL, retinal nerve fiber layer; ONH, optic nerve head; VD, vessel density; SVD, superficial vessel density; DVD, deep vessel density*.

* *Indicates statistically significant difference between the groups. P < 0.05 were considered statistically significant*.

In intergroup comparisons within diabetic eyes without DR (NoDR eyes), those with myopia demonstrated reduced whole VD of ONH comparing to those without myopia(45.61 ± 6.32 vs. 49.91 ± 4.36; 95% CI, 1.95–6.27; *P* < 0.001). Peripapillary VD demonstrated a similar decrease while comparing NoDR eyes with myopia to those without myopia (48.52 ± 6.99 vs. 51.36 ± 4.24; 95% CI, 0.56–5.11; *P* = 0.006) (Figure 1).

**Figure 2:**
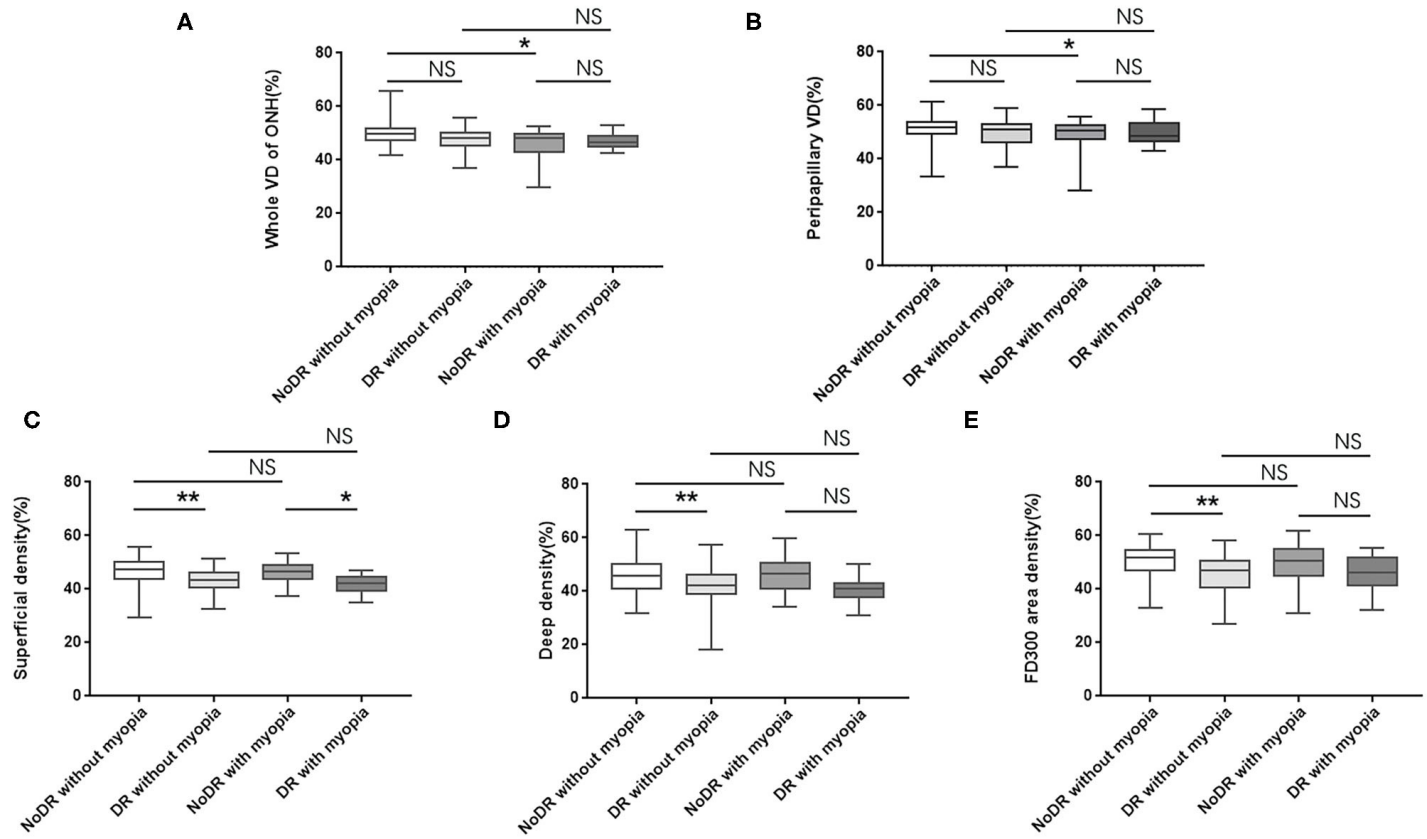
Differences in VD of ONH and macula among subgroups **(A–E)**. VD, vessel density; ONH, optic nerve head; NoDR, diabetic eyes without diabetic retinopathy; DR, eyes with diabetic retinopathy; NS, no significant. *P < 0.05; **P < 0.001. *P* < 0.05 were considered statistically significant.

**Figure 3:**
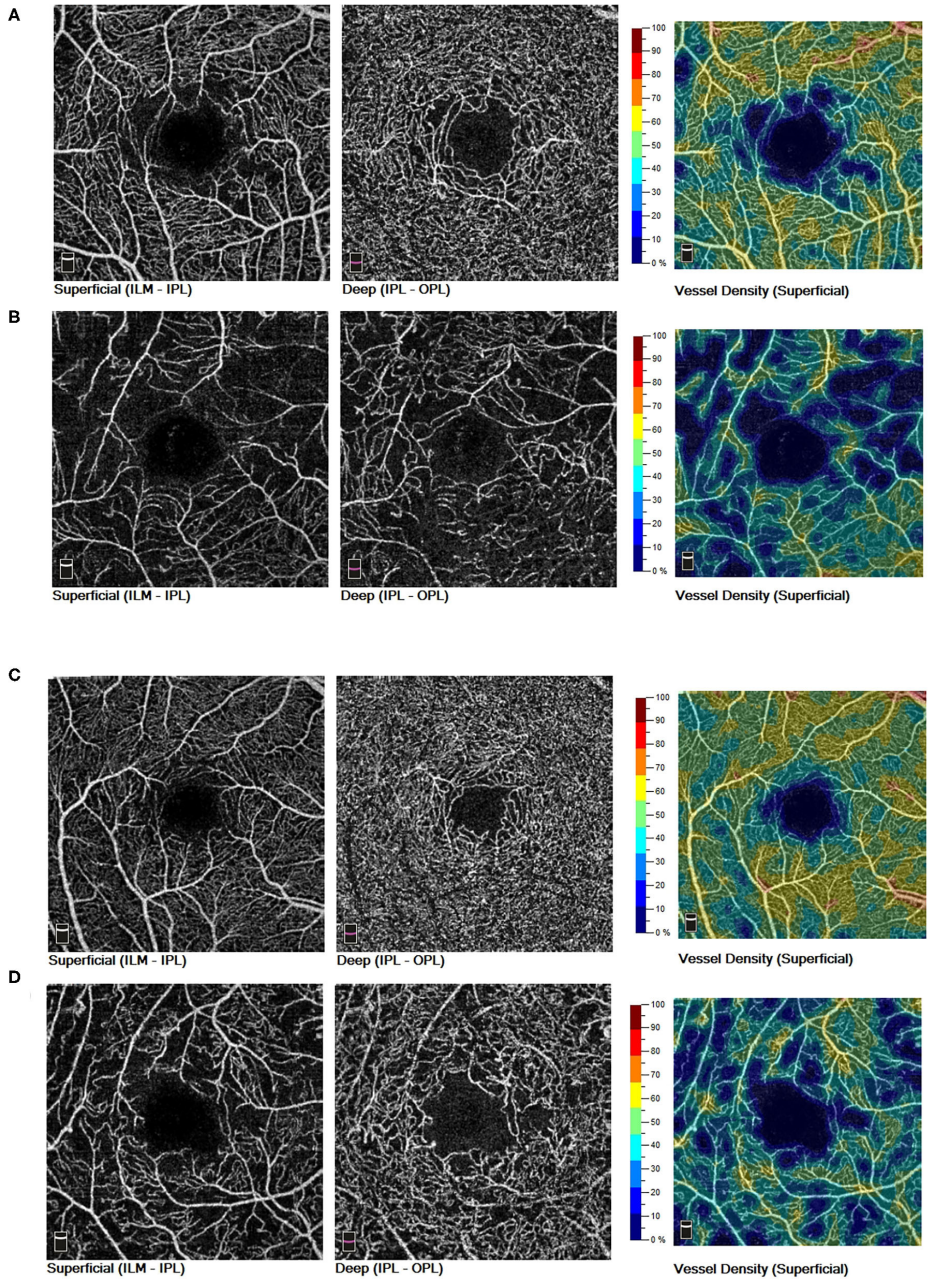
In non-myopic eyes of diabetic patients, both SVD and DVD showed decreased trend from NoDR eyes **(A)** to DR eyes **(B)**. In myopic eyes of diabetic patients, only SVD demonstrated a decreased trend from NoDR eyes **(C)** to DR eyes **(D)**. SVD, superficial vessel density; DVD, deep vessel density; NoDR, diabetes without diabetic retinopathy; DR, diabetic retinopathy.

In intragroup comparison of eyes without myopia, DR eyes showed significant reduction in SVD (43.01 ± 4.25 vs. 46.58 ± 4.90; 95% CI, 1.80–4.61; *P* < 0.001), DVD (42.15 ± 6.31 vs. 45.64 ± 6.34; 95% CI, 1.07–5.01; *P* < 0.001), and FD300 area density (44.95 ± 6.96 vs. 50.31 ± 5.74; 95% CI, 2.88–7.27; *P* < 0.001) while comparing to NoDR eyes. However, in intragroup comparison of diabetic eyes with myopia, only SVD demonstrated decrease (41.68 ± 3.34 vs. 45.99 ± 4.17; 95% CI, 1.10–7.22; *P* = 0.002) in DR eyes while comparing to NoDR ones (Figures 2, 3).

## Discussion

The effect of myopia on VD was complicated. In healthy adults, myopia were reported to decrease the VD in either peripapillary (10, 17), the macula (8), or in both regions (6, 9). The possible mechanism was that excessive elongation of the globe would cause thinning of the retina and result in reduced oxygen demand. Alternatively, the elongation of globe would mechanically stretch retinal tissue, resulting in decreased vascular endothelial growth factor production (18, 19). Both mechanisms would possibly result in reduced retinal microvascular density. The fact that myopic changes were mainly located in the peripapillary area compared to the parafoveal area (20) made peripapillary area more vulnerable to the elongating effect of myopia in healthy controls. In diabetic eyes of our study, only reduced VD of ONH, but not VD of macula, were observed in myopic eyes compared to non-myopic ones. The reason could be that compared to normal individuals, diabetic patients had thinner RNFL (21) which might required less regional oxygen and blood supply in the peripapillary area, resulting in the reduction of VD in peripapillary region via autoregulatory mechanisms (20). The superimposed effect of myopia and diabetes on the reduced RNFL thickness would make the VD of ONH more susceptive than the VD in macular region in diabetic patients.

As demonstrated in our further subgroup analysis, the association of myopia with reduction of VD in ONH were only significant in NoDR eyes, but not in DR eyes. It was suggested that, in NoDR eyes, myopia probably played an independent role in the reduction of VD apart from the effect of diabetes. However, in eyes with DR, myopia played a minor role compared to that of DR, or the deteriorated effect of DR on the VD overwhelmed the protective effect of myopia. The result was consistent with other study, although the researchers did not take myopia or axial length into consideration (22). There were also several studies reporting different results that peripapillary VD of both NoDR and DR patients were lower comparing to VD of normal controls. These discrepancies could be explained by the fact that there were no significant differences in axial length among NoDR, DR, and control groups in the study (23) or that myopia and axial length were not considered while analyzing the OCTA parameters (24–26).

In diabetic eyes with and without DR, the microvascular alterations in macular region varied while considering myopia. Our results suggested that DR were closely associated with decreased SVD, DVD, and FD300 area density in non-myopic eyes; however, in myopic eyes, only SVD exhibited reduction while comparing DR eyes to NoDR eyes. The change of DVD between DR and NoDR eyes in myopic group was not so dramatic as that in non-myopic group, probably implying that myopia could protect the retinal microvasculature from severe damage of DR via DVD. The possible mechanism was that the axial elongation caused retinal neurodysfunction particularly in the outer retina (27), resulting in reduced metabolic demand and consequent decrease in the VD particularly in outer retina. Degenerative changes in the outer retina would therefore increase the facility of oxygen acquisition in inner retina to partly alleviate the hypoxic assaults in outer retina (28) and therefore alleviated the reduction of SVD in diabetes. However, even in myopic eyes, DR were closely related the decrease of SVD, indicating that in myopic eyes, SVD could alternatively serve as a sensitive indicator of microvascular alterations of DR. The effect of myopia could partly explain the conflicting results of other studies in which either SVD or DVD (or both) were decreased in DR (29, 30). Therefore, we also suggested that when analyzing the VD in macular region, axial length, and myopia should be considered.

This study had several limitations. First, a relatively high proportion of younger patients were included in the group of diabetic patients with myopia. Based on a recent study indicating that VD did not change significantly with age (31), the difference between myopic and non-myopic patients was not considered interfering in the study. However, considering other studies showed a significant decrease in VD with age in Chinese population (32), we speculated that the effect of myopia was underestimated. Second, our study shared the limitations of cross-sectional studies that could not confirm causation. The question regarded to whether microvascular alterations in myopic eyes occurred before or after RD remained uncertain. Further prospective longitudinal studies were needed. Third, the SVD would superimpose on the DVD. The artifact was called “projection artifact.” Additionally, the OCTA software could introduce artifacts such as loss of detail, doubling of vessels, stretching defects, or false flow artifact (33). In the present study, we excluded OCTA images with significant projection artifacts and other software-induced artifacts to minimize the interference. Nevertheless, it was impossible to completely avoid artifacts which might inevitably exert some confounding effects on the analyses.

In summary, our findings suggested that in diabetic patients, myopic eyes showed lower DR prevalence and thinner RNFL thickness at the nasal and inferior region. In NoDR period, myopia were closely related to the reduction of whole VD of ONH and peripapillary VD. DR were closely related to the reduction of SVD and DVD in non-myoptic eyes, but were closely related to the reduction of SVD in myopic eyes. When analyzing microvascular alterations in diabetic eyes, axial length, and myopia should be taken into consideration.

## Data Availability Statement

The raw data supporting the conclusions of this article will be made available by the authors, without undue reservation.

## Ethics Statement

The study was approved by the research Ethics Committee of Sun Yat-sen Memorial Hospital, Sun Yat-sen University. The patients/participants provided their written informed consent to participate in this study. Written informed consent was obtained from the individual(s) for the publication of any potentially identifiable images or data included in this article.

## Author Contributions

XW, YZ, and YL: design. XW and ZL: conduct of the study and analysis. XW and XL: collection and preparation. XW, XL, and JX: management. XW, YZ, and JX: interpretation of data. XW, ZL, YZ, and YL: review. XW, ZL, YZ, JX, XL, and YL: approval of the manuscript. All authors contributed to the article and approved the submitted version.

## Funding

This research was financially supported by Natural Science Foundation of Guangdong Province (2015A030313019) and Sun Yat-sen Clinical Research Cultivating Program (SYS-Q-202104). The funders had no role in study design, data collection and analysis, decision to publish, or preparation of the manuscript.

## Conflict of Interest

The authors declare that the research was conducted in the absence of any commercial or financial relationships that could be construed as a potential conflict of interest.

## Publisher's Note

All claims expressed in this article are solely those of the authors and do not necessarily represent those of their affiliated organizations, or those of the publisher, the editors and the reviewers. Any product that may be evaluated in this article, or claim that may be made by its manufacturer, is not guaranteed or endorsed by the publisher.
